# Sweet taste does not modulate pain perception in adult humans

**DOI:** 10.12688/wellcomeopenres.15726.2

**Published:** 2020-08-05

**Authors:** Elizabeth R Mooney, Alexander J Davies, Anthony E Pickering

**Affiliations:** 1 School of Physiology, Pharmacology & Neuroscience, Biomedical Sciences Building, University of Bristol, Bristol, BS8 1TD, UK; 2 Anaesthesia, Pain & Critical Care Sciences, Translational Health Sciences, Bristol Medical School, University of Bristol, Level 7, Bristol Royal Infirmary, Bristol, BS2 8HW, UK; 3 Nuffield Department of Clinical Neuroscience, University of Oxford, Level 6 West Wing, John Radcliffe Hospital, Oxford, OX3 9DU, UK

**Keywords:** Pain, Sucrose, Sweetness, Endogenous Analgesia, Hedonia

## Abstract

**Background**: Sugar is routinely used to comfort neonates undergoing painful procedures, and animal studies have shown that sucrose increases the time to withdrawal from painful stimuli. However, there are no published studies examining the effects of sweet substances on heat pain thresholds and percept in adult humans.

**Methods**: Healthy adult volunteers (n=27, aged 18-48 years) were recruited to a controlled, double-blind, randomised, cross-over study to characterise the effect of tasting solutions of equivalent sweetness (10% sucrose and 0.016% sucralose) on warm detection and heat pain thresholds and the percept ratings of painfully hot stimuli. The effect of anticipation of a sweet taste on heat pain threshold was also assessed.

**Results**: Tasting either sucrose or sucralose had no significant effect on the percept of an individually titrated hot stimulus (54.5±4.2 and 54.9±3.2 vs 53.2±3.5 for water, 0-100 visual analogue scale), on the warm detection or heat pain threshold (43.3±0.8, 43.2±0.8 vs 43.0±0.8°C). Anticipation of a sweet substance similarly did not affect heat pain thresholds.

**Conclusions**: Sucrose and sucralose solutions had no analgesic effect when assessed using heat detection thresholds and percept ratings of painfully hot stimuli despite being perceived as sweeter and more pleasant than water. These findings are in contrast to results reported from previous animal studies in which thermal analgesia from sweet solutions is robust. Given the ubiquitous availability of sugar rich drinks in the modern environment, the lack of observable effect may be due to an insufficient hedonic value of the test solutions when compared to the experience of a laboratory rodent. Alternatively, sweet tastes may have a specific effect on pain tolerance rather than the threshold and acute percept measures assayed in this study.

## Introduction

Sugar is in routine clinical use in the neonatal intensive care setting to comfort neonates undergoing painful procedures ^1, 2^. A number of studies have demonstrated that sucrose has an analgesic effect in immature rodents, particularly in neonatal rats, acting to increase the thermal sensory threshold ^3, 4^. A recent study has demonstrated that active consumption of sucrose also produces an analgesic effect in adult rats ^5^, slowing withdrawal from a painful thermal stimulus. This rapid, robust and transient effect was observed when the rats actively consumed the sucrose and also after training when it was provided passively. The analgesic effect was not prevented by opioid, noradrenaline or dopamine antagonists but was blocked by cannabinoid CB1 receptor antagonists. The analgesic effect was seen with artificial sweeteners and also seen when rats anticipated a sweet solution but were provided with water.

A limited number of studies have examined the effect of sweet tastes on pain thresholds in human adults and children ^6, 7^. These studies suggest that sucrose can alter both the perception and tolerance of pain, although there are some suggestions that this is true only in males ^8^ or in individuals with a preference for sugary tastes ^6^. Studies in adult humans have largely used tolerance of cold pain as an assessment: a suprathreshold test which is both unpleasant and known to act as a generalised stressor ^9^, making these studies less comparable to the rodent studies which have typically assessed pain thresholds using escapable heat stimuli. Furthermore, factors behind the anticipation of sweet and rewarding substances have not been studied in the context of pain sensation.

Therefore, we aimed to develop a simple assay to assess the analgesic effect of sweet taste in adult humans. We examined the effects of sweet taste on the perception and tolerance of painful thermal stimuli in adults. We used both calorific (sucrose) and zero-calorie (sucralose) sweetened solutions alongside a neutral control liquid (water) and assessed their effects on the detection of thermal stimuli, threshold for painful heat stimuli and the pain felt on delivery of a calibrated hot stimulus (i.e. to assess stimulus and percept locking). The effect of anticipation of sweet taste on thermal detection and tolerance was also investigated. In contrast to previous results in the adult rat, our findings did not demonstrate an equivalent analgesic effect of sweet taste in adult humans. We discuss our results with reference to the existing literature on the phenomenon of sweet taste or sucrose analgesia.

## Methods

The study was approved by University of Bristol Faculty of Biomedical Science Research Ethics Committee (reference 60062).

### Population

A power calculation was undertaken for the main outcome measure of the effect of sweet taste on pain intensity in response to a standardised thermal stimulus established at baseline for each subject as being ~60/100mm on a VAS scale. We expect to see a 15% reduction in that pain score based on previous studies which would be biologically and clinically meaningful. The variance of the pain scores was expected to be 12mm (SD, based on data from Brooks *et al.,* 2017 ^10^). With alpha of 0.05 and beta of 0.9 this gives a sample size of 18 subjects for repeated measures testing (G*power).

Healthy volunteer subjects were recruited between December 2017 and March 2018 for entry to the study using poster and email advertisements (see *Extended data*) ^11^ at the University of Bristol. A total of 27 participants (22F:5M) aged 18–48 years took part in the assessments (see *Underlying data* for demographics) ^11^. After participants had expressed an interest in the study, they were sent a participant information leaflet by email (see *Extended data*) ^11^. Participation in the study was precluded by the presence of acute or chronic pain, a neurological disease, a diagnosed medical or psychiatric condition, diabetes or impaired glucose tolerance, use of recreational drugs, use of analgesic medications within the preceding 48 hours, or pregnancy. One participant was excluded due to their disclosing a diagnosis of depression and anxiety (see participant flow diagram in *Extended data*) ^11^.

### Enrolment

Those who wished to proceed then attended a single session which incorporated completion of an inclusion questionnaire (see *Extended data*) ^11^, provision of informed written consent for study entry and the use of any data for research (see consent form in *Extended data*) ^11^ and data collection. The session took place in a purpose-built consultation room within the Clinical Research and Imaging Centre at the University of Bristol, at a date and time agreed with participants. Sessions were conducted by ERM (who is a female medical doctor) and no other staff members were present during the experiments. Sessions lasted between 1 and 1.5 hours each. Subjects were told that the objective of the study was to examine how the sensation of pain interacts with sweet flavours – they were not explicitly told the underlying study hypothesis regarding sweet-taste induced analgesia. 

### Calibration

Participants were asked to rate their current level of thirst on a visual analogue scale (VAS) consisting of a 100mm line labelled with “Not thirsty at all” at one end to “Extremely thirsty” at the other ^12^. Participants marking the scale at a level greater than 30mm were encouraged to drink water to quench their thirst, and then repeated the rating. This was repeated up to three times until the value was below 30mm. Four participants who continued to record a thirst score >30mm despite free access to water were later excluded from analysis due to concerns that thirst may confound the results. Sensitivity analysis with inclusion of these participants did not demonstrate any difference in analgesic effects.

Participants were seated comfortably at a table in a temperature-controlled room with their non-dominant arm resting supinated on a soft support on the table-top. A contact thermode (30×30mm, Medoc TSA-II, Israel) was positioned on the volar forearm (C6 dermatome) and secured in place by a Velcro strap. All instructions to participants were standardised and read from a script (provided as *Extended data*) ^11^. For quantitative sensory testing (QST), the wording of the instructions was standardised in accordance with the DFNS (German Research Network on Neuropathic Pain) protocol ^13^. Warm detection threshold (WDT) was assessed by applying a temperature ramp at 1°C.s ^-1^ from a baseline of 32°C (chosen as the average temperature of the immediately exposed forearm: ^14^) and participants were instructed to press a button when they first felt the thermode becoming warm. Heat pain threshold (HPT) was assessed by applying an identical temperature ramp with the instruction to press the button when the warm sensation changed to painful. After an initial familiarisation trial assessment of WDT and HPT, baseline measurements were taken by applying two temperature ramps in short succession separated by 6s at 32°C ^15^ with the participants instructed to indicate their WDT on ramp 1 and HPT on ramp 2. This was repeated three times at 2 min intervals and mean values calculated.

For the fixed stimulus rating (FSR) experiment, the thermode temperature was incremented using a pseudo-random sequence to identify a stimulus level that produced a pain rating of 60mm on a 100mm pain VAS using the method of limits ^16^. Thermode temperature increased at a rate of 4°C/s and was held at the fixed temperature for 5s. The pain rating was provided by making a mark on a 100mm line anchored with the words “Not painful at all” at the left-hand end and “Extremely painful” at the right-hand end. A new blank scale was presented for the application of the next stimulus. Four participants who did not achieve a rating >50mm even at the maximum temperature of 48°C were excluded from analysis of the FSR component.

### Taste test

Following this initial calibration QST, participants blindly rated the sweetness and pleasantness of the test solutions. Three test solutions used were selected to enable a comparison between calorific and non-calorific sweet substances. The index solution of 10% sucrose was chosen as a concentration equivalent to that commonly contained in sweet beverages ^17^ and sucralose was used as a sweet-tasting, non-calorific control at a concentration (0.016%) selected to be equivalently sweet to 10% sucrose ^18^. Weaker solutions of 5% sucrose and 0.008% sucralose were also prepared to assess comparative pleasantness. Sucrose was purchased as caster sugar (Silver Spoon) from a supermarket and food-grade sucralose (Bulk Powders) was purchased online. Both were diluted using potable tap water and the same tap water was used as a neutral control liquid. Fresh solutions were made up on each testing day. For each test a 10–15ml sample of liquid (comfortable volume determined individually) was held in the mouth for 10s, after which the participant spat out the solution and rinsed their mouth with water.

Each participant rated the solutions by making marks on two 100mm VAS lines, one for sweetness from “Not sweet at all” to “Extremely sweet”, and the other reflecting pleasantness marked “Neutral” to “Extremely pleasant” on the right. Participants were instructed to leave the second scale blank and instead mark a box provided if they found the solution at all unpleasant (‘Dislike’). This assessment was repeated at the end of the test session after all the pain assessments in order to ensure that the reward value of the solutions had not changed significantly.

### Test protocol

On each test day, the experimenter made up the solutions as above and numbered them, making a record of the number allocated to each solution. The flasks were then given to an independent researcher in the university department who re-labelled the flasks (such that the original label was not visible) and documented the re-allocated numbers in a file to which the experimenter had no access until all data collection sessions were complete. Prior to the beginning of the data collection period, a random number generator was used to generate 30 random sequences of the appropriate numbers with which solutions were labelled, and each participant was allocated to a sequence in order of date and time of attendance.

To allow assessment of the influence of sweet taste on thermal thresholds participants were given a plastic cup containing a solution (10% sucrose, 0.016% sucralose or water), which they conveyed to their mouth using their dominant hand. Solutions were presented randomly in blocks of three, with both participant and observer blinded to the identity of the solutions, as described above. Warm detection and heat pain threshold were assessed while participants held 10–15ml of the solution in their mouth. The two-ramp thermal protocol was used to measure WDT followed by HDT. After completion, participants expelled the solution and rinsed their mouth as desired. Each solution was presented three times giving a total of nine trials.

A similar protocol was used to assess the effect of the test solutions on the FSR. The solutions were again presented blind in block randomised order as described above. The calibrated heat stimulus was delivered at the individually pre-determined temperature for 5s, after which the participant marked the 100mm pain VAS.

To assess the effects of anticipation of a sweet solution on WDT and HPT, participants were told that the heat ramp experiment would be repeated first with no solution, then three times with the same sweet solution. The two-ramp protocol was delivered as described above, first in the absence of solution, then repeated with samples of 10% sucrose, water, and 10% sucrose, in a fixed order. One participant who was unable to comply with instructions was excluded from analysis of the anticipation experiment.

### Data analysis

Data is presented as mean ± standard error of the mean (SEM). Statistical tests were performed in Prism 7.0 ( GraphPad). The taste preferences and effects of solution on thermal percept were assessed with repeated measures ANOVA with Bonferroni post-hoc test. Linear regression analysis was used to assess the influence of reward on the change in pain ratings.

## Results

### Participant demographics

A total of 27 participants (22F:5M) aged 18–48 years took part in the assessments. See flow chart ( *Extended data*) ^11^ for details regarding number of participants included in each analysis.

### Sucrose and sucralose are equally sweet and rewarding

**Figure 1.**
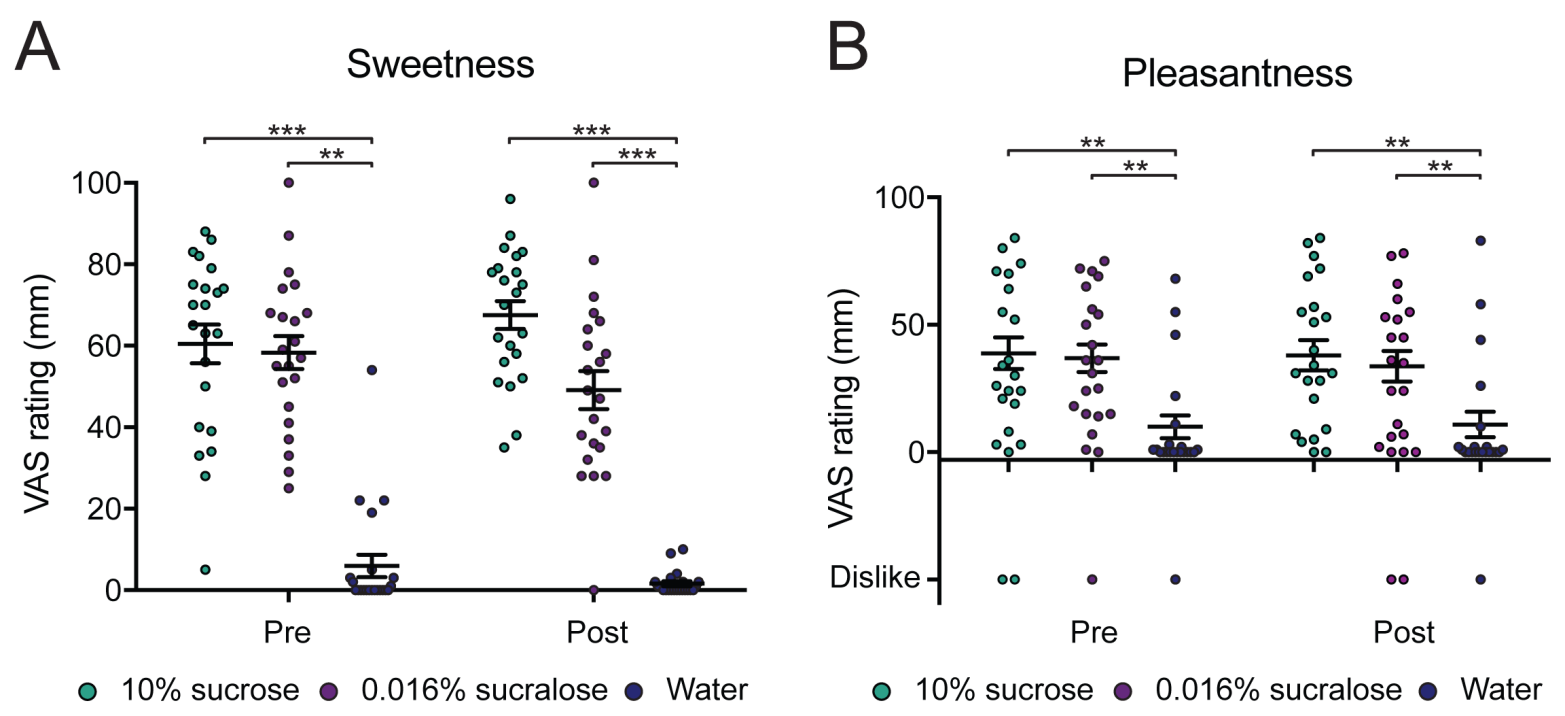
The sweetness and pleasantness of the solutions before and after the experiment. **A**. Sucrose and sucralose were found to be equally sweet on both initial (sucrose 60.5±4.7mm, sucralose 58.3±4.0mm) and final assessments (sucrose 67.5±3.5mm, sucralose 49.1±4.6mm), and both were significantly sweeter than water. **B**. Sucrose and sucralose were perceived to be equally pleasant on both initial (sucrose 38.9±6.2mm, sucralose 37.0±5.4mm) and final assessments (sucrose 38.1±5.9mm, sucralose 35.1±5.8mm). Water was significantly less pleasant than sucrose or sucralose at both assessments. Individuals rating the solutions as unpleasant (‘Dislike’) were excluded from the analysis. There was no significant change in the sweetness or pleasantness ratings of sucrose, sucralose or water between the beginning and end of the experiment (pre vs post). (*** - p<0.001; ** - p<0.01, RM-ANOVA with Bonferroni post hoc tests, n=22). VAS, visual analogue scale.

Sucrose (10%) and sucralose (0.016%) were perceived as equally sweet both at the start and the end of the experiment ( Figure 1A) and both were sweeter than water. Likewise, both sucrose and sucralose were perceived to be equally pleasant on both initial and subsequent assessments and both were more pleasant than water ( Figure 1B). Several participants rated solutions as unpleasant (three for sucrose, three for sucralose and two for water) and so did not provide pleasantness VAS ratings (marked ‘Dislike’).

A previous study in humans demonstrated an analgesic effect of sweet solutions only in those who preferred high concentration sweet solutions ^6^. To assess whether there was a population of participants who had a preference for less sweet solutions, participants also rated the sweetness and pleasantness of weaker solutions of 5% sucrose and 0.008% sucralose. 5% sucrose was rated to be significantly less sweet than 10% sucrose but the difference in sweetness rating between 0.016% and 0.008% sucralose did not reach significance. Both the sweet solutions were rated as significantly sweeter than water. Analysis of the pleasantness ratings of the solutions demonstrated that 10% sucrose was rated as significantly more pleasant than water, although not significantly more pleasant than 5% sucrose. The same was true of high and low concentrations of sucralose. There was no clear separation into populations who preferred low to high concentrations of sweet solution. Given these results, only the higher concentrations of sucrose (10%) and sucralose (0.016%) were used in all subsequent tests. Tap water was used as a neutral, non-rewarding control solution.

### Sweet taste has no effect on heat pain perception

**Figure 2.**
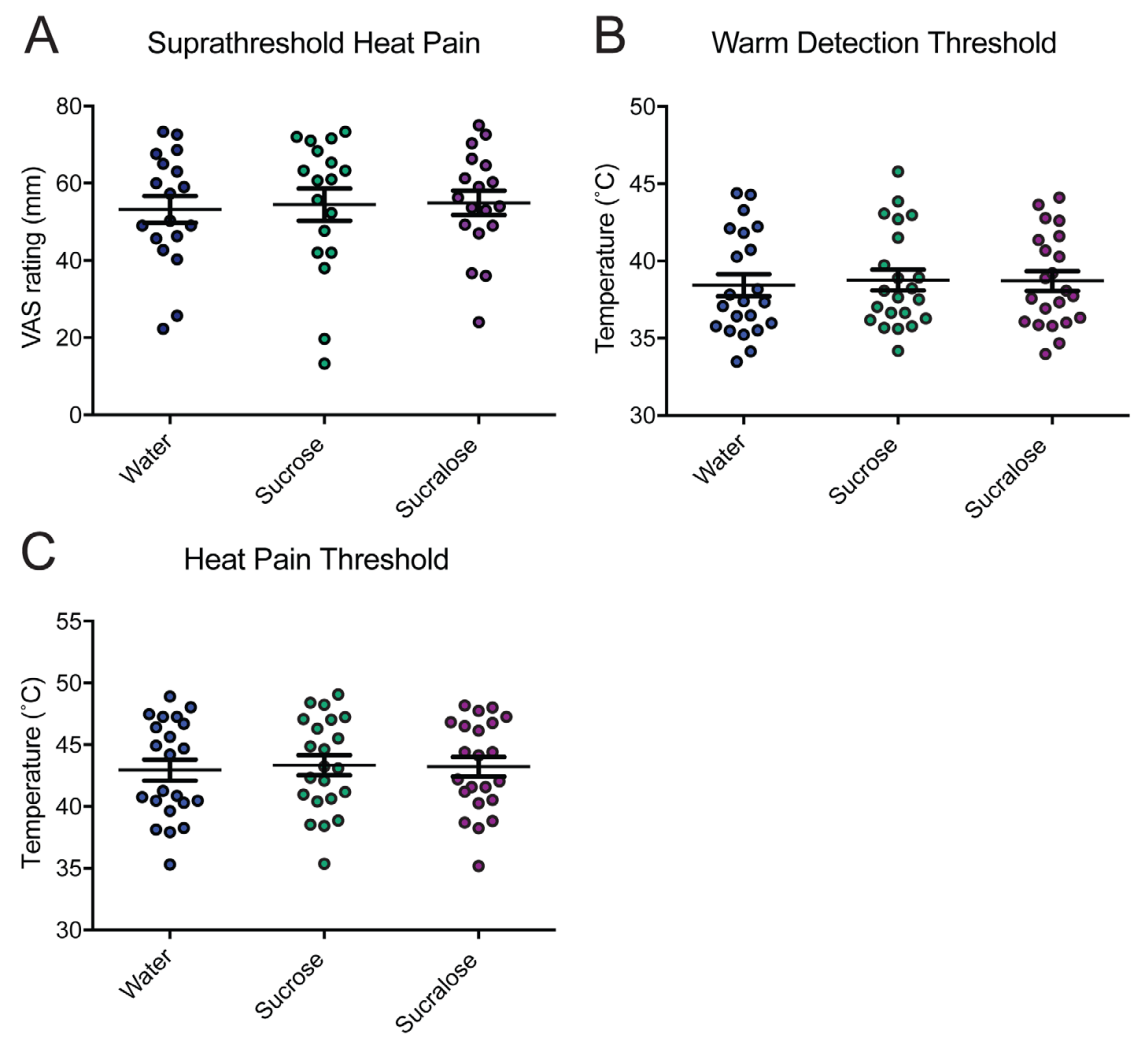
Effect of sweet taste on fixed stimulus rating, warm detection and heat pain thresholds. **A**: The mean visual analogue scale (VAS) pain rating when presented with a heat stimulus which had previously produced a rating of 60mm in the absence of solution. There were no differences between the VAS in the presence of 10% sucrose, 0.016% sucralose or water (p>0.05, n=18). **B**: No difference was observed in the warm detection threshold in the presence of 10% sucrose, 0.016% sucralose or water (p>0.05, n=22). **C**: There was no significant difference in the mean heat pain threshold recorded in the presence of 10% sucrose (43.3±0.8°C), 0.016% sucralose (43.2±0.8°C) or water control (43.0±0.8°C).

The effects of the test solutions on the perceived painfulness (assessed by VAS score) of the calibrated stimulus (FSR) was assessed while the participant held test solution in their mouth. There was no significant difference in the FSR in the presence of 10% sucrose, 0.016% sucralose or water control ( Figure 2A). This suggests that sweet taste did not affect the perceived painfulness of a calibrated thermal stimulus.

### Sweet taste has no effect on warm detection threshold or heat pain threshold

The effects of sweet taste on the WDT and HDT were assessed consecutively while participants held test solution in their mouth. There was no significant difference between WDT or HPT measured in the presence of 10% sucrose, 0.016% sucralose or water control ( Figure 2B, 2C).

### Anticipation of a sweet taste does not affect thermal sensitivity

Animal studies have demonstrated that rats trained to anticipate sucrose show an increase in thermal withdrawal latency when they are presented with water instead of sucrose ^5^, suggesting that the anticipation of sucrose alone is sufficient to affect thermal sensitivity. In order to investigate this in human subjects, we told participants that they would receive the same sweet solution on three occasions. They were then presented first with sucrose, then with water, and subsequently with sucrose again. The protocol was designed such that an effect of sucrose anticipation on thermal sensitivity would be revealed by the presence of an increased heat pain threshold on presentation of water.

**Figure 3.**
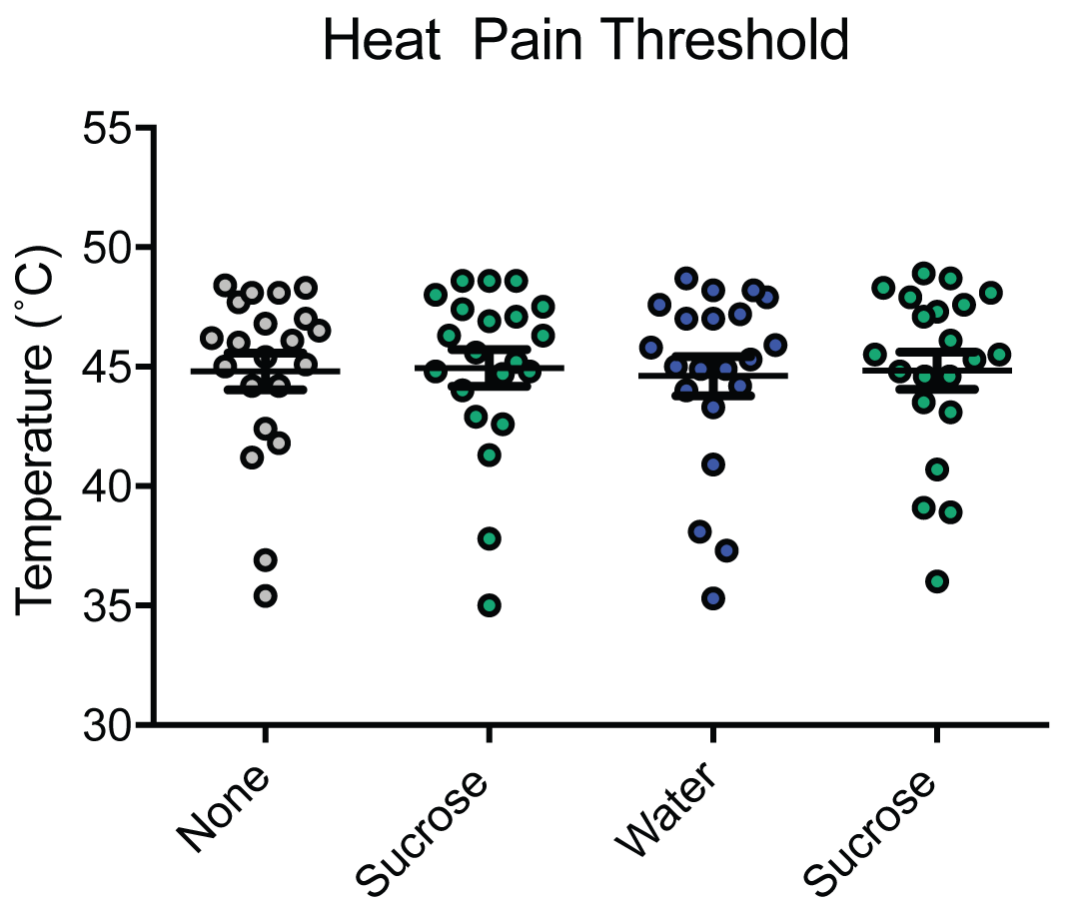
Anticipation of sucrose does not alter heat pain thresholds. The mean heat pain thresholds recorded at baseline (44.8±0.8°C) and on presentation of 10% sucrose (45.0±0.8°C), water (44.6±0.8°C) and 10% sucrose (44.8±0.8°C) in sequence (p>0.05, n=21).

At the start of this experimental phase, warm detection and heat pain thresholds were re-assessed in the absence of any solution and subsequent measurements in the presence of solutions were compared to these baselines. There was no significant difference between the HPT at baseline and on the first presentation of sucrose. There was no significant change in heat pain threshold on presentation of water when sucrose was anticipated, and subsequent presentation of sucrose did not lead to a significant change in thresholds. These results demonstrate that anticipation of sucrose did not have an analgesic effect, regardless of whether or not sucrose is received ( Figure 3).

### Inter-individual perception of sweetness or pleasantness does not influence pain

**Figure 4.**
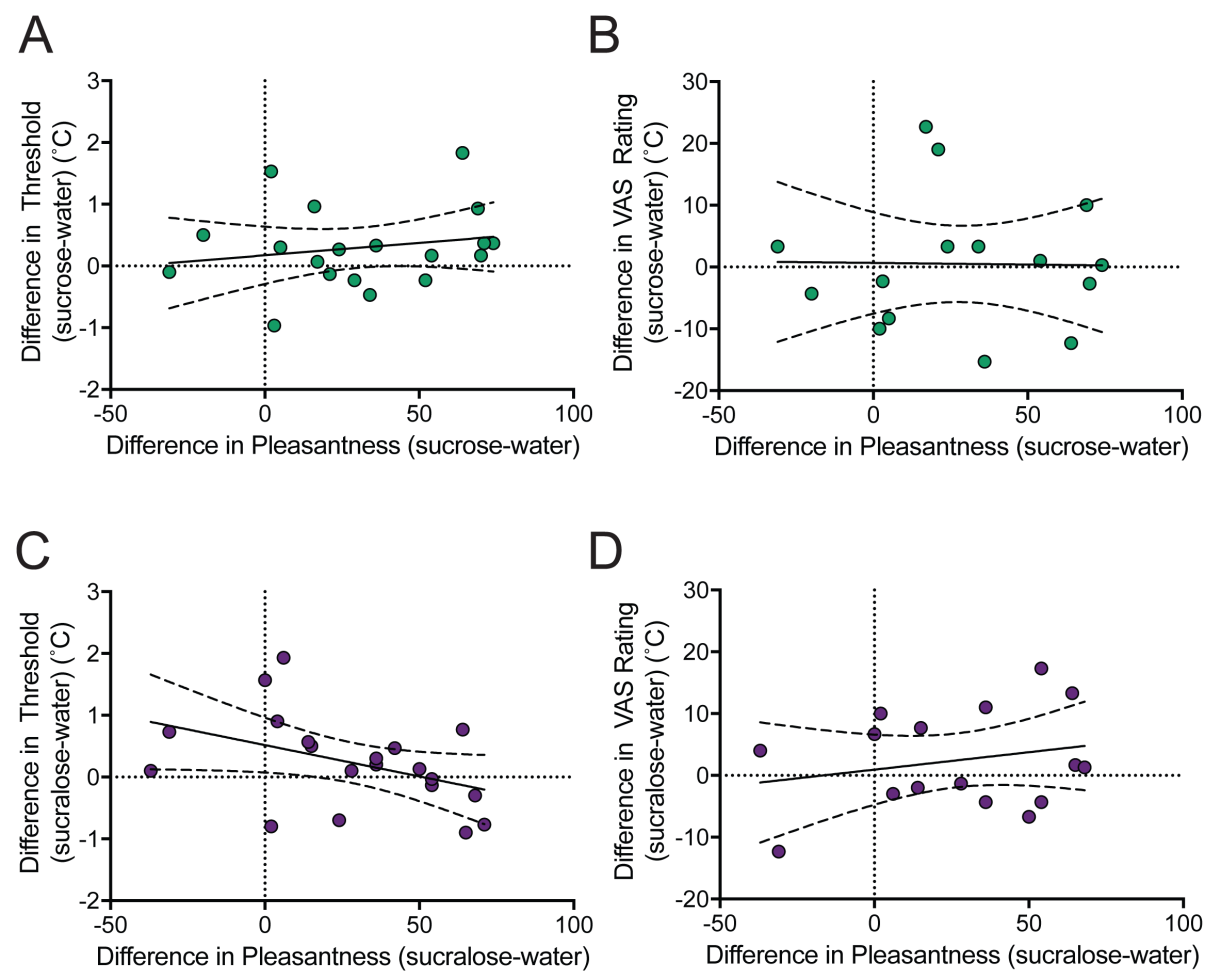
Relationship between pleasantness of test solutions and heat pain. **A**: The difference in heat pain thresholds measured in the presence of 10% sucrose and water (control) plotted against the difference in pleasantness for each individual (linear regression r2=0.036, ns, n=19). **B**: The difference in fixed stimulus rating reported in the presence of 10% sucrose and water plotted against the difference in pleasantness for each individual (linear regression, r2=0.0002, ns, n=15). **C**: The difference in heat pain thresholds measured in the presence of 0.016% sucralose and water control plotted against the difference in pleasantness for each individual (linear regression, r2=0.18, ns, n=20). **D**: The difference in fixed stimulus rating reported in the presence of 10% sucrose and water control plotted against the difference in pleasantness for each individual (linear regression, r2=0.05, ns, n=16). Graphs show regression lines with 95% confidence intervals (dotted). VAS, visual analogue scale.

The pleasantness VAS ratings of the sweet solutions in comparison to water showed considerable variation across individuals (from -31 to +74mm for 10% sucrose and -31 to +71mm for 0.016% sucralose, n=19). Regression analysis was used to assess whether there was any correlation between the relative pleasantness of solutions and their analgesic effect ( Figure 4). There was no significant correlation between the relative pleasantness rating of 10% sucrose and the difference in heat pain threshold or the fixed stimulus rating (r ^2^=0.036 and 0.0002, respectively) ( Figure 4A and 4B). There was no significant correlation between the relative pleasantness rating of 0.016% sucralose and the change in FSR (r ^2^=0.05) ( Figure 4C and 4D). There was an unexpected and weak negative relationship between relative pleasantness rating of 0.016% sucralose and difference in heat pain threshold, though the slope of the regression line failed to reach statistical significance (r ^2^=0.18, p=0.06, Figure 4C). Overall these results demonstrate that there is no positive association between the pleasantness of any sweet solution and its effect on pain perception.

## Discussion

It is commonly observed that humans who are in a negative emotional state seek solace in the form of sweet foods and drinks ^19^. Recent evidence further suggest a relationship between pain suffering and emotional eating that is driven by anxiety sensitivity ^20, 21^ and chronic pain has been associated with an increased prevalence of eating disorders in young people ^22^. Studies in rats show that, similarly to humans, highly palatable foods are sought in response to stress ^23^, or anxiety ^24^, and suggest that limited intake of sucrose may in fact dampen the physiological responses to stress ^[Bibr ref-23], 25^. We have previously, shown in adult rats that a sucrose solution is sufficient to elicit short term thermal analgesia during consumption ^5^. We therefore posed the question of whether a similar neurophysiological phenomenon to sweet taste may occur in adult humans.

Contrary to our initial hypothesis, we found no convincing effect of sweet taste on thermal pain perception in our study. The possible reasons for the absence of an effect, which is in contrast to previous studies in both humans and rodents, are discussed below.

### Hedonic value

A possible mechanism by which sweet flavours affect pain sensation is via the reward/hedonic pathway. While the interaction between chronic pain and affect has been extensively described and discussed ^26, 27^, there are also reports of modulation of subjective perception of acute pain and even of nociceptive withdrawal reflexes by pleasant stimuli including images and odours ^28, 29^. These effects have been attributed to activation of limbic structures which exert modulatory effects on nociceptive signals at a spinal level ^30^. A previous study investigating the effects of sweet flavours on pain perception suggested that the reward or hedonic value of the sweet substance was important, as an analgesic effect was only seen in individuals who had a preference for the sweeter solutions ^6^.

Studies in adult rats have demonstrated that both chocolate and sucrose had an analgesic effect in the naïve state, but not after induction of nausea or in the context of a conditioned aversion to sucrose ^31^. Furthermore, it has been shown that a solution which does not normally have a positive hedonic value or analgesic effect, in this case sodium chloride, does have an analgesic effect when the solution becomes desirable in the context of sodium depletion ^32^. These studies suggest that it is the reward value, rather than the specific taste or the pharmacological properties of the solution, which confers the analgesic effect. 

In our study, the majority of participants reported the test solutions to be pleasant, and there was no correlation between an individual’s pleasantness rating and pain perception. In comparison to the homologous rodent experiment ^5^, the hedonic value of a sugar solution may be considerably less to modern humans who live in a sugar rich environment than to a rat raised on a monotonous chow diet. Thus, it is possible that holding a simple sugar/sweetener solution in the mouth, although rated as pleasant, did not have sufficient reward value to produce an analgesic effect in adult humans ^[Bibr ref-33]^. Use of highly palatable sweet food or prior mild water deprivation may instead be required to observe a significant analgesic effect ^34^.

### Modality of pain stimulus

Most previous studies demonstrating sucrose-induced analgesia in humans have used the cold pressor test ^6, 8, 35, 36^. Analgesic effects on pressure pain have also been observed in adult humans during consumption of sweet and highly palatable food or drink ^34^. Our study shows that the analgesic phenomenon does not – at least in the context presented to participants in our study - appear to extend to modulation of thermal sensitivity and heat pain thresholds or percept.

Different models of pain and hyperalgesia are sensitive to modulation by specific classes of exogenous analgesics (e.g. opioids, NMDA receptor antagonists ^37, 38^). The modality of pain sensation affected by sucrose or hedonic consumption is therefore likely to depend on the mechanism of endogenous analgesia. While sweet taste analgesia in neonatal animals is thought to depend on endogenous opioid signalling ^39– 41^, analgesia from hedonic drinking in adult animals is thought to require endocannabinoid signalling ^5^. Data from human newborns are equivocal but suggest a non-opioid mechanism ^42, 43^. Thus, engagement of differing neurotransmitter systems and endogenous analgesic pathways by the various reported sucrose analgesia paradigms may variably affect different nociceptive modalities.

### Motivation and affect

Unlike measurements of pain thresholds (i.e. nociception), cold pain tolerance as measured by the cold pressure test reflects a broader psychological response to pain and is confounded by stress ^9, 44^. A study examining cold perception in young adults while holding a 24% sucrose solution in their mouth observed an analgesic effect on cold pain tolerance but not sensory thresholds ^36^. Similarly, changes to pressure pain tolerance but not pressure threshold were also observed in adults ^34^. Sweet taste in adults may therefore preferentially modify the motivational or affective aspects of the pain experience, as revealed in assays of pain tolerance, rather than the threshold and acute pain percept (nociceptive) measures tested in the current study. Further investigation of the phenomenon using assays for thermal pain tolerance ^45^ would therefore be of interest.

In concordance with previous human studies ^6, 8, 35, 36^, participants in the current study were asked to hold the sweet solutions in their mouths during the test stimulus. The lack of a motivational component in our tasting assay, as well as the cognitive demand on participants to hold the solutions in their mouths without drinking, could also help explain some of the discrepancy with the previous study in adult rats in which the animals were required to actively seek and consume the sweet solution ^5^.

A concentration of 24% sucrose is typically recommended prior to heel lancing and venepunctures in neonates ^2^, and this concentration was reported as effective in the cold pressor task of pain tolerance in adult humans ^36^. It is therefore possible that a thermal analgesic effect could have been seen had we used a higher concentrations (>24%) of sucrose in our participants. In our study, we chose a sucrose concentration of 10% (0.29M) as that most commonly used in sweetened beverages ^46^, and within the range of concentrations associated with the highest liking (0.21M-0.3M sucrose) ^47^. It is noteworthy that two participants in the current study rated 10% sucrose as ‘unpleasant’. 0.25M to 0.5M sucrose (8.6% to 17.2%) is considered a flexion point in the ‘liking’ rating, such that concentrations of sucrose above this are characterised by a decrease in liking by ‘sweet dislikers’, which make up around 20% of adults aged 18–34 ^47^. Therefore, increasing the sweetness of the taste solution would require stratification of the volunteer group by sweet preference to eliminate the confound of aversion in such sweet ‘dislikers’ ^47^.

### Influence of age

Some animal studies have reported that analgesic effects of passive sucrose are seen only in neonatal rats ^39^, whereas studies in adult rats have demonstrated a clear analgesic effect with active consumption ^5, 31, 48^, suggesting an age-dependence on the context in which sucrose analgesia is apparent. Similarly, in humans an effect was demonstrated only in children and not in adults ^6^. In children, the analgesic effect of sweet taste was restricted to cold thresholds but not tolerance to cold ^49^. It is therefore possible that purely anti-nociceptive effects of sucrose are only present in neonatal and immature humans, which may account for the lack of effect in this adult population.

### Power and Sex

A limitation of our study is the relatively low number of participants. Like previous studies in adults ^8, 36^ our experiments were powered to detect differences in thermal thresholds of 15% or more (a decrease of >9mm on a VAS score). While it is possible that smaller but still clinically relevant differences (<15%) between groups may be detectable if more participants were included in the study. However, the effect size detected in our investigation (of an increase in pain VAS of ~1mm with sucrose/sucralose) does not provide any evidence of a clinically meaningful effect.

Sex differences in the phenomenon have also been described in humans; for example, the analgesic effect of sucrose in the cold pressor test was observed in adult males but not females ^6, 8, 35^. On the other hand, sweet taste analgesia in an assay of pressure pain tolerance in adults revealed differences in only female participants, a finding attributed to a greater preference for sweets in women than men ^34^. In rats, a significant effect of sucrose on thermal sensitivities was seen in both sexes ^5^. Although our study included both male and female participants and an exploratory sensitivity analysis of the two populations independently did not alter our findings, the low proportion of male participants (5 versus 22 female) means that our study is not sufficiently powered to detect a sex-specific differences.

## Conclusion

In conclusion, our study has demonstrated no clear effect of sweet-tasting solutions (either calorific or non-calorific) on the perception of warm temperature or heat pain in adult humans. This finding differs from some previously reported studies in humans and observations made in rodents. Potential explanations for the discrepancy include that the experimental solutions used lacked sufficient hedonic value for the study participants. Differences in the modality of sensory testing and motivational component of the taste assay may also contribute to variability in observations of the sucrose analgesia phenomenon. We hypothesise that alternative substances such as commercial sweet drinks or chocolate, which have a stronger association with pleasure than our clear, unbranded sugary liquid, may have a more substantial have a more substantial hedonic value and therefore greater potential analgesic effect. Further work is therefore required to investigate the effects of sucrose and sweet taste on human sensory physiology.

## Data availability

### Underlying data

Data.Bris: Mooney 2020 WellcomeOpenRes. https://doi.org/10.5523/bris.11ruhu8y19oxe2ihn9qb11igk3 ^11^

This project contains the following underlying data:

- Fig1A_Sweetness.csv
- Fig1B_Pleasantness.csv
- Fig2A_Suprathreshold.csv
- Fig2B_WDT.csv
- Fig2C_HPT.csv
- Fig3_HPT.csv
- Fig4.csv
- sucrose_data_mastersheet.xlsx

### Extended data

Data.Bris: Mooney 2020 WellcomeOpenRes. https://doi.org/10.5523/bris.11ruhu8y19oxe2ihn9qb11igk3 ^11^

This project contains the following extended data:

- Sucrose study documents.pdf (participant information leaflet, inclusion questionnaire, consent form, email and poster advertisements, participant instructions script)
- Participants.pdf (participant flow chart)

Data are available under the terms of the Creative Commons Attribution 4.0 International license (CC-BY 4.0).
