# Supplementary material for: Sweet taste does not modulate pain perception in adult humans
Source: Wellcome Open Res. 2020 Aug 5;5:43. Originally published 2020 Mar 11. [Version 2] doi: 10.12688/wellcomeopenres.15726.2 (PMC7361508; doi:10.12688/wellcomeopenres.15726.2)
Supplement: Peer review history

# Peer review: Reviewer response for version 2

**DOI:** 10.21956/wellcomeopenres.17677.r39837

**Published:** 2020-08-11

**Authors:** Steve Davidson

**Affiliations:** 1 Department of Anesthesiology and Pain Research Center, University of Cincinnati, College of Medicine, Cincinnati, OH, USA

The revisions and sidebar discussions are appropriate.

Is the work clearly and accurately presented and does it cite the current literature?

Yes

If applicable, is the statistical analysis and its interpretation appropriate?

Yes

Are all the source data underlying the results available to ensure full reproducibility?

Yes

Is the study design appropriate and is the work technically sound?

Yes

Are the conclusions drawn adequately supported by the results?

Yes

Are sufficient details of methods and analysis provided to allow replication by others?

Yes

Reviewer Expertise:

Neurobiology of pain

I confirm that I have read this submission and believe that I have an appropriate level of expertise to confirm that it is of an acceptable scientific standard.

# Peer review: Reviewer response for version 1

**DOI:** 10.21956/wellcomeopenres.17239.r39255

**Published:** 2020-07-14

**Authors:** Naomi J Meesters

**Affiliations:** 1 Department of Pediatrics, Division of Neonatology, Erasmus MC-Sophia Children’s Hospital, Rotterdam, The Netherlands

This study focuses on the effect of sweet taste on heat pain threshold in adults. The study method is clearly and extensively described. Many of the factors that could have influenced the pain threshold are taken into account.

 I mainly focused on a comparison with the existing evidence regarding this subject in neonates. I have some concerns/questions regarding the methods that were used to study the effect of pain threshold in adults:

1. Even though a power calculation was performed, I wonder if the expected 15% reduction on the VAS scale is realistic. In contrast to analgesics, there are no (or only minor) negative consequences related to the administration of sweet tasting solutions. Therefore a smaller reduction in pain score could also be clinically relevant.
2. It was already mentioned in the introduction that a difference between males and females was described. Therefore I do not understand why it was chosen to include 22 female and 5 males. In the discussion it was described that and exploratory sensitivity analysis did not show any difference finding, but I wonder if this is reliable considering the low number of males that were included.
3. For neonates, a much higher concentration of sucrose is studied most often and used in clinical care (24%). Why was a concentration of 10% used in this study? It is explained that it was used because of it’s equivalence to sweet beverages, but at this point it is unclear if the solution did not reduce pain threshold because it was not sweet enough or if it can not reduce pain threshold in general.
4. Another difference with neonatal studies is the way the sweet solution was administered. The underlying mechanisms of the pain reducing effect of sucrose in neonates are not fully understood and possibly multifactorial. While in neonates the sucrose will be ingested, in this study the participants were instructed to hold the liquid in the mouth.
5. In neonates often a waiting period (mostly two minutes) is used between the administration of sucrose and the painful procedure. In this study, the measurement of the pain threshold was performed during the experience of tasting the sweet solutions. It is not known if it might take a certain period before the sweet tasting has an effect on the pain threshold.

Is the work clearly and accurately presented and does it cite the current literature?

Yes

If applicable, is the statistical analysis and its interpretation appropriate?

Yes

Are all the source data underlying the results available to ensure full reproducibility?

Yes

Is the study design appropriate and is the work technically sound?

Yes

Are the conclusions drawn adequately supported by the results?

Yes

Are sufficient details of methods and analysis provided to allow replication by others?

Yes

Reviewer Expertise:

Neonatal pain.

I confirm that I have read this submission and believe that I have an appropriate level of expertise to confirm that it is of an acceptable scientific standard.

# Response

**Published:** 2020-07-28

**Authors:** Alexander Davies

**Affiliations:** University of Oxford, UK

**Reviewer 3**

**Reviewer:** *This study focuses on the effect of sweet taste on heat pain threshold in adults. The study method is clearly and extensively described. Many of the factors that could have influenced the pain threshold are taken into account.*

*I mainly focused on a comparison with the existing evidence regarding this subject in neonates. I have some concerns/questions regarding the methods that were used to study the effect of pain threshold in adults:*

*Even though a power calculation was performed, I wonder if the expected 15% reduction on the VAS scale is realistic. In contrast to analgesics, there are no (or only minor) negative consequences related to the administration of sweet tasting solutions. Therefore a smaller reduction in pain score could also be clinically relevant.*

**Authors:**  We agree with the reviewer that a smaller reduction in pain score could still be clinically relevant and have added the following statement to the discussion:

“A limitation of our study is the relatively low number of participants. Like previous studies in adults (Lewkowski et al., 2003; Kakeda & Ishikawa, 2011) our experiments were powered to detect differences in thermal thresholds of 15% or more (a decrease of >9mm on a VAS score). While it is possible that smaller but still clinically relevant differences (<15%) between groups may be detectable if more participants were included in the study.  However, the effect size detected in our investigation (of an increase in pain VAS of ~1mm with sucrose/sucralose) does not provide any evidence of a clinically meaningful effect.”

**Reviewer:** *It was already mentioned in the introduction that a difference between males and females was described. Therefore I do not understand why it was chosen to include 22 female and 5 males. In the discussion it was described that and exploratory sensitivity analysis did not show any difference finding, but I wonder if this is reliable considering the low number of males that were included.*

**Authors:**     An equal ratio of sexes, although desired, was not prospectively specified. Participation was on a voluntary basis and owing to limitations on the length of the study recruitment was stopped having reached 27 individuals. We agree that balanced sexes would be preferable to identify sex-specificity, and in this regard the study is underpowered. We have added the following sentence to the discussion:

“…the low proportion of male participants (5 versus 22 female) mean our study is not sufficiently powered to detect a sex-specific differences.”

**Reviewer:** *For neonates, a much higher concentration of sucrose is studied most often and used in clinical care (24%). Why was a concentration of 10% used in this study? It is explained that it was used because of it’s equivalence to sweet beverages, but at this point it is unclear if the solution did not reduce pain threshold because it was not sweet enough or if it can not reduce pain threshold in general.*

**Authors:**     We agree with the reviewer that 10% sucrose may not be sweet enough to increase the pain threshold. However, we also caution the use of very high sucrose concentrations owing to the possibility of sweetness aversion in a subset of volunteers. We have therefore added the following to the discussion:

A concentration of 24% sucrose is typically recommended prior to heel lancing and venepunctures in neonates (Stevens et al., 2004), and this concentration was reported as effective in the cold pressor task of pain tolerance in adult humans (Lewkowski et al., 2003). It is therefore possible that a thermal analgesic effect could have been seen had we used a higher concentrations (>24%) of sucrose in our participants. In our study, we chose a sucrose concentration of 10% (0.29M) as that most commonly used in sweetened beverages (Ventura et al., 2011), and within the range of concentrations associated with the highest liking (0.21M-0.3M sucrose (Iatridi et al., 2019). It is noteworthy that two participants in the current study rated 10% sucrose as ‘unpleasant’. 0.25M to 0.5M sucrose (8.6% to 17.2%) is considered a flexion point in the ‘liking’ rating, such that concentrations of sucrose above this are characterised by a decrease in liking by ‘sweet dislikers’, which make up around 20% of adults aged 18-34 (Iatridi et al., 2019). Therefore, increasing the sweetness of the taste solution would require stratification of the volunteer group by sweet preference to eliminate the confound of aversion in such sweet ‘dislikers’ (Iatridi et al., 2019).

**Reviewer:** *Another difference with neonatal studies is the way the sweet solution was administered. The underlying mechanisms of the pain reducing effect of sucrose in neonates are not fully understood and possibly multifactorial. While in neonates the sucrose will be ingested, in this study the participants were instructed to hold the liquid in the mouth.*

**Authors:** We agree that the neonatal phenomenon is likely to be multifactorial. Therefore, in this study we explicitly set out to examine the requirement for sweet taste without the possible confound ingestion. Our results point to sweet taste alone being insufficient for sucrose’s potential analgesic effect.  We also acknowledge discrepancies between this study and previous animal experiments in which sucrose analgesia is observed:

     “The lack of a motivational component in our tasting assay, as well as the cognitive demand on participants to hold the solutions in their mouths without drinking, could also help explain some of the discrepancy with the previous study in adult rats in which the animals were required to actively seek and consume the sweet solution”.

**Reviewer:** *In neonates often a waiting period (mostly two minutes) is used between the administration of sucrose and the painful procedure. In this study, the measurement of the pain threshold was performed during the experience of tasting the sweet solutions. It is not known if it might take a certain period before the sweet tasting has an effect on the pain threshold.*

**Authors:** The rationale for the short duration between pairs of stimuli was to directly compare with the rat experiments in which analgesia was observed within seconds of the onset of sucrose consumption. Equally, the analgesic phenomenon in rats appeared to extinguish fairly quickly after cessation of consumption (Davies et al., 2019). We therefore think it unlikely for a delayed analgesic effect to appear beyond the tasting period.

The short testing duration also reduced the burden on participants holding the test solutions in their mouths for long periods of time. However, we agree that longer duration tasting and/or sucrose concentration may be required to observe a measurable effect.

We thank the reviewer for their time and their careful and considered assessment our work. 

**References for reviewer 3**

Davies AJ, Kim D, Park J, Lee JY, Vang H, Pickering AE & Oh SB. (2019). Hedonic drinking engages a supraspinal inhibition of thermal nociception in adult rats. Pain 160, 1059-1069.

Iatridi V, Hayes JE & Yeomans MR. (2019). Quantifying Sweet Taste Liker Phenotypes: Time for Some Consistency in the Classification Criteria. Nutrients 11.

Kakeda T & Ishikawa T. (2011). Gender differences in pain modulation by a sweet stimulus in adults: A randomized study. Nursing & Health Sciences 13, 34-40.

Lewkowski MD, Ditto B, Roussos M & Young SN. (2003). Sweet taste and blood pressure-related analgesia. Pain 106, 181-186.

Stevens B, Yamada J & Ohlsson A. (2004). Sucrose for analgesia in newborn infants undergoing painful procedures. Cochrane Database Syst Rev, CD001069.

Ventura EE, Davis JN & Goran MI. (2011). Sugar content of popular sweetened beverages based on objective laboratory analysis: focus on fructose content. Obesity (Silver Spring) 19, 868-874.

# Peer review: Reviewer response for version 1

**DOI:** 10.21956/wellcomeopenres.17239.r39254

**Published:** 2020-07-14

**Authors:** Steve Davidson

**Affiliations:** 1 Department of Anesthesiology and Pain Research Center, University of Cincinnati, College of Medicine, Cincinnati, OH, USA

This is a clearly executed and written study to examine whether sweet taste alters the perception of threshold warm and hot stimuli. The conclusion, supported by the data, is that sweet taste does not alter thermal heat perception in adult humans. The study was carefully designed, although limited in scope. 

 A few issues reduce the overall impact of the study which should be addressed. 

1. The study was designed to identify differences from the mean pain score of 15% in the treatment group. This is a highly ambitious change in threshold perception given that many of the best analgesics do not perform this well. Therefore, the study may not have been adequately powered to find smaller (more likely) differences, i.e., the limited number of subjects tested may have prevented finding a smaller, but true difference. 
2. The number of male subjects to female subjects (about a 1:4 ratio) prevents meaningful inference of the interpretation of sex differences in this study, a point of interest which would have been worth the effort. Gender and sex are not synonyms. 
3. There are a few speculative statements in the study without adequate citation or literature support including:
  a) that humans seek sugary foods during pain
  b) living in a sugar rich environment dampened the potential analgesic effects of this study. 
  c) commercial drinks and chocolate are not valid for comparison because of many additional odors and flavors compared to sugar water. The goal of this study is not to test whether reward alters pain thresholds (otherwise cocaine would be just as appropriate) - it is specifically to test sweet taste. 
4. In the animal studies, and perhaps some of the previous human studies, subjects may have been allowed to actually consume the sweet food/drink, however in this study subjects had to hold it in their mouths during testing and then spit it out. This may have produced the unwanted consequence of high cognitive demand to resist the desire of consumption, potentially confounding the interpretation. 
5. Pain tolerance is discussed, although not tested in this study. There is no discussion of pain tolerance in animal studies, to parallel the discussion on threshold. 
6. The timing between heating stimulus rounds may have been too short. What is the rationale for choosing this protocol? 

Is the work clearly and accurately presented and does it cite the current literature?

Yes

If applicable, is the statistical analysis and its interpretation appropriate?

Yes

Are all the source data underlying the results available to ensure full reproducibility?

Yes

Is the study design appropriate and is the work technically sound?

Yes

Are the conclusions drawn adequately supported by the results?

Yes

Are sufficient details of methods and analysis provided to allow replication by others?

Yes

Reviewer Expertise:

Neurobiology of pain

I confirm that I have read this submission and believe that I have an appropriate level of expertise to confirm that it is of an acceptable scientific standard.

# Response

**Published:** 2020-07-28

**Authors:** Alexander Davies

**Affiliations:** University of Oxford, UK

**Reviewer 2**

*This is a clearly executed and written study to examine whether sweet taste alters the perception of threshold warm and hot stimuli. The conclusion, supported by the data, is that sweet taste does not alter thermal heat perception in adult humans. The study was carefully designed, although limited in scope.*

**

*A few issues reduce the overall impact of the study which should be addressed.*

*1.* **Reviewer:** *The study was designed to identify differences from the mean pain score of 15% in the treatment group. This is a highly ambitious change in threshold perception given that many of the best analgesics do not perform this well. Therefore, the study may not have been adequately powered to find smaller (more likely) differences, i.e., the limited number of subjects tested may have prevented finding a smaller, but true difference.*

**Authors:** Our power calculation was based on differences observed in previous similar studies. Lewkowski et al identified a change in cold tolerance of more than 18% when participants held sweet solutions in their mouths (Lewkowski *et al.*, 2003). Kakeda & Ishikawa observed a 19% increase in pain threshold time in the cold pressor test in men with sucrose water held in their mouths relative to distilled water (Kakeda & Ishikawa, 2011). We agree that further participants would be required to detect smaller differences. As addressed below, increasing the number of males specifically would address the question of sex-specificity in the proposed analgesic effect seen in other studies. We have added the following sentences to the discussion:

“A limitation of our study is the relatively low number of participants. Like previous studies in adults (Lewkowski et al., 2003; Kakeda & Ishikawa, 2011) our experiments were powered to detect differences in thermal thresholds of 15% or more (a decrease of >9mm on a VAS score). While it is possible that smaller but still clinically relevant differences (<15%) between groups may be detectable if more participants were included in the study.  However, the effect size detected in our investigation (of an increase in pain VAS of ~1mm with sucrose/sucralose) does not provide any evidence of a clinically meaningful effect.”

*2.* **Reviewer:** *The number of male subjects to female subjects (about a 1:4 ratio) prevents meaningful inference of the interpretation of sex differences in this study, a point of interest which would have been worth the effort. Gender and sex are not synonyms.*

**Authors:** We agree that sex is the correct term to be use and this has been updated in the revised manuscript. An equal ratio of sexes, although desired, was not prospectively specified. Participation was on a voluntary basis and, owing to limitations on the length of the study recruitment period, was stopped having reached 27 individuals. We agree that balanced sexes would be preferable to identify sex-specificity, and in this regard the study is underpowered. We have added the following sentence to the discussion:

“…the low proportion of male participants (5 versus 22 female) mean our study is not sufficiently powered to detect a sex-specific differences.”

*3.* **Reviewer:** *There are a few speculative statements in the study without adequate citation or literature support including:*

*a) that humans seek sugary foods during pain*

**Authors:** Evidence in humans suggests positive associations between sweet, palatable food-seeking/consumption and depression (Westover & Marangell, 2002; Knuppel *et al.*, 2017), anxiety (Penaforte *et al.*, 2019) and chronic stress (Tryon *et al.*, 2013), all of which are frequently co-morbid with chronic pain (Attal *et al.*, 2011; Woda *et al.*, 2016; Sieberg *et al.*, 2018).

Anecdotal evidence suggests chronic pain sufferers seek solace in the form of hedonic consumption (Janke & Kozak, 2012), which may be driven indirectly through anxiety-triggered sensitivity for emotional eating (Janke *et al.*, 2016; Kauffman *et al.*, 2019). Chronic pain has also been associated with an increased prevalence of eating disorders in young people, and may include over-eating as well as under-eating conditions (Sim *et al.*, 2017). Obesity, for example, is often co-morbid with chronic pain (Okifuji & Hare, 2015). Chronic pain patients also show increased gustatory sensitivity to sweet substances (Small & Apkarian, 2006), suggesting changes to brain circuity that may make them more susceptible to sweet foods.

Negative emotions and stress may pre-dispose for the consumption of sweet and palatable foods with the aim of relieving the aversive state (Gibson, 2006). Studies in rats also show that highly palatable foods are sought in response to a stressful (Ulrich-Lai *et al.*, 2011), or anxiolytic state (Ghitza *et al.*, 2006), and suggest that limited intake of sucrose may in fact dampen the physiological responses to stress (Ulrich-Lai *et al.*, 2010; Ulrich-Lai *et al.*, 2011).

We agree that in its simplicity the statement ‘humans seek sugary foods during pain’ could be misleading and that the evidence for a link between chronic pain and sugary foods is more nuanced. We have therefore removed this statement from the abstract and introduction, and moved to the discussion where we have made the following changes to better reflect on the speculative nature of the association:

“It is commonly observed that humans who are in a negative emotional state seek solace in the form of sweet foods and drinks (Gibson, 2006). Recent evidence further suggest a relationship between pain suffering and emotional eating that is driven by anxiety sensitivity (Janke et al., 2016; Kauffman et al., 2019) and chronic pain has been associated with an increased prevalence of eating disorders in young people (Sim et al., 2017). Studies in rats show that, similarly to humans, highly palatable foods are sought in response to stress (Ulrich-Lai et al., 2011), or anxiety (Ghitza et al., 2006), and suggest that limited intake of sucrose may in fact dampen the physiological responses to stress (Ulrich-Lai et al., 2010; Ulrich-Lai et al., 2011)."

*b)* **Reviewer:** *living in a sugar rich environment dampened the potential analgesic effects of this study.*

**Authors:** This statement on access to sweet foods (comparing lab rodents to humans) is not in doubt. However, we agree that we have no evidence that this played a role in damping the human analgesic response to sweet taste – this is speculative rather than evidence based but we prefer to keep it in the discussion as an interesting hypothesis. The main point however, is that sucrose solution alone is relatively less rewarding than the complex nutrition and flavour of sweet foods typically consumed by humans. We have therefore removed this sentence and cite recent evidence that foods high in fat and carbohydrate are more rewarding, calorie for calorie, than foods high only in fat or carbohydrate (DiFeliceantonio *et al.*, 2018).

*c)* **Reviewer:** *commercial drinks and chocolate are not valid for comparison because of many additional odors and flavors compared to sugar water. The goal of this study is not to test whether reward alters pain thresholds (otherwise cocaine would be just as appropriate) - it is specifically to test sweet taste.*

**Authors:** We have of course taken a reductive approach to the question of whether sucrose, so often a component of highly palatable foods, is sufficient to elicit thermal analgesia in adult humans as it appears to do in neonates and in rats. However, we feel it is important to place the study into context with real life, in which sugar is purposely consumed with additional flavours and odours, likely due to the greater reward obtained from consumption of these foods as outlined above. It will be interesting therefore for future studies to investigate whether more nutritionally complex palatable food stuffs are sufficiently rewarding to elicit thermal analgesia using a similar protocol. We have modified the final paragraph to reflect this potential for future work:

“We hypothesise that alternative substances such as commercial sweet drinks or chocolate, which have a stronger association with pleasure than our clear, unbranded sugary liquid, may **have a more substantial hedonic value and therefore greater potential** analgesic effect.”

*4.* **Reviewer:** *In the animal studies, and perhaps some of the previous human studies, subjects may have been allowed to actually consume the sweet food/drink, however in this study subjects had to hold it in their mouths during testing and then spit it out. This may have produced the unwanted consequence of high cognitive demand to resist the desire of consumption, potentially confounding the interpretation.*

**Authors:** This is an interesting point and would again emphasise the cognitive nature of sucrose reward mediating any potentially analgesic effect rather than taste perception alone. We have added a sentence to the discussion:

“The lack of a motivational component in our tasting assay, **as well as the cognitive demand on participants to hold the solutions in their mouths without drinking,** could also help explain some of the discrepancy with the previous study in adult rats in which the animals were required to actively seek and consume the sweet solution”.

*5.* **Reviewer:** *Pain tolerance is discussed, although not tested in this study. There is no discussion of pain tolerance in animal studies, to parallel the discussion on threshold.*

**Authors:** The valency afforded to pain tolerance renders it susceptible to cognitive factors (Cimpean & David, 2019). Therefore, we hypothesise that thermal pain tolerance, rather than merely pain intensity (threshold), may be preferentially affected by sweet consumption, as has been shown for cold pain tolerance in humans (Lewkowski *et al.*, 2003).

Owing to the subjective nature of pain and suffering, one must be cautious in attempting to ascribe the phenomenon of pain tolerance to animals. Indeed, a change in thermal withdrawal latencies in rats could indicate either a change in nociceptive threshold or an increased tolerance of a suprathreshold stimuli. Without verbal confirmation of the pain threshold we are not able to distinguish these effects. Furthermore, eliciting pain in animals without the ability to escape the painful stimulus would be unethical. We have therefore restricted our discussion to human studies, and instead add the following proposal for further work:

“Further investigation of the phenomenon using assays for thermal pain tolerance (Lue et al., 2018) would therefore be of interest.”

*6.* **Reviewer:** *The timing between heating stimulus rounds may have been too short. What is the rationale for choosing this protocol?*

**Authors:** The rationale for the short duration between pairs of stimuli was to directly compare with the rat experiments in which analgesia was observed within seconds of the onset of sucrose consumption. The short testing duration also reduced the burden on participants to hold the test solutions in their mouths for long periods of time. Intervals of 4-6 s between thermal stimuli has previously been validated as producing reproducible thermal thresholds in normal subjects (Wasner & Brock, 2008), therefore we do not expect this to confound our results. We have added this reference to the methods section.

We thank the reviewer for taking the time to thoughtfully assess our work and are grateful for their feedback, which has helped to greatly improve the paper.

**References for reviewer 2**

Attal N, Lanteri-Minet M, Laurent B, Fermanian J & Bouhassira D. (2011). The specific disease burden of neuropathic pain: results of a French nationwide survey. Pain 152, 2836-2843.

Cimpean A & David D. (2019). The mechanisms of pain tolerance and pain-related anxiety in acute pain. Health Psychol Open 6, 2055102919865161.

DiFeliceantonio AG, Coppin G, Rigoux L, Edwin Thanarajah S, Dagher A, Tittgemeyer M & Small DM. (2018). Supra-Additive Effects of Combining Fat and Carbohydrate on Food Reward. Cell Metab 28, 33-44 e33.

Ghitza UE, Gray SM, Epstein DH, Rice KC & Shaham Y. (2006). The anxiogenic drug yohimbine reinstates palatable food seeking in a rat relapse model: a role of CRF1 receptors. Neuropsychopharmacology 31, 2188-2196.

Gibson EL. (2006). Emotional influences on food choice: sensory, physiological and psychological pathways. Physiol Behav 89, 53-61.

Janke EA, Jones E, Hopkins CM, Ruggieri M & Hruska A. (2016). Catastrophizing and anxiety sensitivity mediate the relationship between persistent pain and emotional eating. Appetite 103, 64-71.

Janke EA & Kozak AT. (2012). "The more pain I have, the more I want to eat": obesity in the context of chronic pain. Obesity (Silver Spring) 20, 2027-2034.

Kakeda T & Ishikawa T. (2011). Gender differences in pain modulation by a sweet stimulus in adults: A randomized study. Nursing & Health Sciences 13, 34-40.

Kauffman BY, Rogers AH, Bakhshaie J, Mayorga NA, Garza M, Ochoa-Perez M, Lemaire C & Zvolensky MJ. (2019). Examining the Relationship Between Pain Intensity and Emotional Eating Among Latinos in a Federally Qualified Health Center: The Role of Anxiety Sensitivity. J Immigr Minor Health 21, 1217-1223.

Knuppel A, Shipley MJ, Llewellyn CH & Brunner EJ. (2017). Sugar intake from sweet food and beverages, common mental disorder and depression: prospective findings from the Whitehall II study. Sci Rep 7, 6287.

Lewkowski MD, Ditto B, Roussos M & Young SN. (2003). Sweet taste and blood pressure-related analgesia. Pain 106, 181-186.

Okifuji A & Hare BD. (2015). The association between chronic pain and obesity. J Pain Res 8, 399-408.

Penaforte FRO, Minelli MCS, Anastacio LR & Japur CC. (2019). Anxiety symptoms and emotional eating are independently associated with sweet craving in young adults. Psychiatry Res 271, 715-720.

Sieberg CB, Taras C, Gomaa A, Nickerson C, Wong C, Ward C, Baskozos G, Bennett DLH, Ramirez JD, Themistocleous AC, Rice ASC, Shillo PR, Tesfaye S, Edwards RR, Andrews NA, Berde C & Costigan M. (2018). Neuropathic pain drives anxiety behavior in mice, results consistent with anxiety levels in diabetic neuropathy patients. Pain Rep 3, e651.

Sim LA, Lebow J, Weiss K, Harrison T & Bruce B. (2017). Eating Disorders in Adolescents With Chronic Pain. J Pediatr Health Care 31, 67-74.

Small DM & Apkarian AV. (2006). Increased taste intensity perception exhibited by patients with chronic back pain. Pain 120, 124-130.

Tryon MS, Carter CS, Decant R & Laugero KD. (2013). Chronic stress exposure may affect the brain's response to high calorie food cues and predispose to obesogenic eating habits. Physiol Behav 120, 233-242.

Ulrich-Lai YM, Christiansen AM, Ostrander MM, Jones AA, Jones KR, Choi DC, Krause EG, Evanson NK, Furay AR, Davis JF, Solomon MB, de Kloet AD, Tamashiro KL, Sakai RR, Seeley RJ, Woods SC & Herman JP. (2010). Pleasurable behaviors reduce stress via brain reward pathways. Proc Natl Acad Sci U S A 107, 20529-20534.

Ulrich-Lai YM, Ostrander MM & Herman JP. (2011). HPA axis dampening by limited sucrose intake: reward frequency vs. caloric consumption. Physiol Behav 103, 104-110.

Wasner GL & Brock JA. (2008). Determinants of thermal pain thresholds in normal subjects. Clin Neurophysiol 119, 2389-2395.

Westover AN & Marangell LB. (2002). A cross-national relationship between sugar consumption and major depression? Depress Anxiety 16, 118-120.

Woda A, Picard P & Dutheil F. (2016). Dysfunctional stress responses in chronic pain. Psychoneuroendocrinology 71, 127-135.

# Peer review: Reviewer response for version 1

**DOI:** 10.21956/wellcomeopenres.17239.r38378

**Published:** 2020-06-09

**Authors:** Richard Hulse

**Affiliations:** 1 School of Science and Technology, Nottingham Trent University, Nottingham, UK

This study performed by Mooney and colleagues investigates the impact taste, focussing upon sweet taste, has upon pain perception. A large body of literature presents that by influencing taste pathways such as via sucrose and sweet taste can influence pain. A number of differing experimental approaches including human and animal studies have elucidated an analgesic capacity for sugar consumption. This is widely acknowledged in the clinical setting such as sugar administration to infants whom undergo painful clinical procedures. This study targets an adult human setting to evaluate how sweet taste may influence the perception of heat induced sensory experiences targeting both perception and tolerance of heat induced pain. However, how sweet taste can influence differing modalities and the fundamental neural axis’ associated with sensory perception is underrepresented in the article, especially in the context of heat pain in adults. Could the authors provide an expansion of the included literature to provide a broader introduction to the study, providing scope for further discussion?

 Interestingly the article initially presents itself with the context that the human population widely seek sugar in times of ‘pain or discomfort seek solace in the form of sweet foods and drinks’. This statement highlights a phenomenon that is of significant importance. It is widely appreciated that pain is a fundamental physiological protective mechanism, as well as being a significant burden to the clinical setting inclusive of the patient, carer and healthcare systems. Additionally, during these times modern dietary composition and mental health are under the spotlight. Can this initial statement become expanded with additional literature to support how the authors see this study fitting into a wider research landscape. For example, is there data available to highlight increased sugar dietary consumption in pain patients or possible association with pain treatments or other consumed drugs? Consideration of the utilisation and benefit of using sweet taste would also be appreciated in the introduction to allow a greater audience to appreciate this work.

 An aspect of the study is to elucidate whether there is a disconnect between heat pain perception and tolerance. The justification of this; both as anatomical and neuronal function was tentatively outlined in the introduction, an aspect of this was provided in the discussion as hedonic/reward via the limbic system. Could a detailed expansion of this be provided as currently ‘modulatory’ actions is used as a term and putative mechanisms of actions would be appreciated by the reader?

 The proposed methodology and general experimental design were appropriate, with overall structure well considered.

 Comments

- In reference to the dose of sugar given; in the discussion it was highlighted that young adults in ref 26 were given a higher concentration of sucrose to induce analgesia. Could an aspect of discussion include reasoning around why a 10% dosing was utilised for this study, and how this differing dose may have implications in the data presented in this study. Also what dose is given in the clinic?
- 32°C used as a baseline why was this chosen? Room temp is usually 20-25. Does this negate detection thresholds?

- 6 seconds between heating ramps? Is this sufficient time to provide recovery both dissipation of heat from the skin and also neuronal activity? For example skin temperature takes time to return to normal temperature post stimulation with varying temperature ramps whether hot or cold (McMullan 2004 ^1^, Hulse 2012 ^2^). Additionally, how the sensory afferents respond to these ‘rapid fire’ temperature spikes i.e. induction of nociceptor sensitisation/desensitisation.

- Why was second dose of less concentrated sugar data not presented? Could this be included?
- Figure 3 warm detection threshold test performed and mentioned but not presented. Could this be incorporated?
- A sentence in discussion could possibly be revised as it suggests that this article identifies an antinociceptive affect, which it did not. Recommendation to delete *italic* text. 
  ‘It is therefore possible that purely antinociceptive effects of sucrose, *as measured by thermal sensitivity in our study,* are only present in neonatal and immature humans, which may account for the lack of effect in this adult population.’

Is the work clearly and accurately presented and does it cite the current literature?

Yes

If applicable, is the statistical analysis and its interpretation appropriate?

Yes

Are all the source data underlying the results available to ensure full reproducibility?

Yes

Is the study design appropriate and is the work technically sound?

Yes

Are the conclusions drawn adequately supported by the results?

Yes

Are sufficient details of methods and analysis provided to allow replication by others?

Yes

Reviewer Expertise:

Neurobiology, pain.

I confirm that I have read this submission and believe that I have an appropriate level of expertise to confirm that it is of an acceptable scientific standard.

## References

[1] : A reliable method for the preferential activation of C- or A-fibre heat nociceptors. *J Neurosci Methods*.2004;138(1-2) : 10.1016/j.jneumeth.2004.03.020 133-9 10.1016/j.jneumeth.2004.03.020 15325121

[2] : Differential roles of galanin on mechanical and cooling responses at the primary afferent nociceptor. *Mol Pain*.2012;8: 10.1186/1744-8069-8-41 41 10.1186/1744-8069-8-41 22672616PMC3404965

# Response

**Published:** 2020-06-30

**Authors:** Alexander Davies

**Affiliations:** University of Oxford, UK

**Responses to Reviewer**

The authors would like to thank the Reviewer for their time and careful assessment of our manuscript. We provide answers to each of the points raised by the Reviewer in the text below.

***Reviewer: **This study performed by Mooney and colleagues investigates the impact taste, focussing upon sweet taste, has upon pain perception. A large body of literature presents that by influencing taste pathways such as via sucrose and sweet taste can influence pain. A number of differing experimental approaches including human and animal studies have elucidated an analgesic capacity for sugar consumption. This is widely acknowledged in the clinical setting such as sugar administration to infants whom undergo painful clinical procedures. This study targets an adult human setting to evaluate how sweet taste may influence the perception of heat induced sensory experiences targeting both perception and tolerance of heat induced pain. However, how sweet taste can influence differing modalities and the fundamental neural axis’ associated with sensory perception is underrepresented in the article, especially in the context of heat pain in adults. Could the authors provide an expansion of the included literature to provide a broader introduction to the study, providing scope for further discussion?*

**Author response:** A reduction in nocifensive responses to both thermal and mechanical stimuli by intraoral sucrose (7.5%) has been demonstrated in neonatal rats (Anseloni *et al.*, 2002). The endogenous analgesia pathway in neonates may involve a descending inhibitory mechanism (Anseloni *et al.*, 2005), potentially suppressing both sensory modalities at the spinal level. In this study we set out specifically to test the effect of sweet taste on thermal sensory thresholds in adults. This had been reported in animal studies but there were no data using a thermal assay in humans. Additionally, thermal stimulation (using a heat ramp device) represents a convenient method to apply a well-controlled stimulus of defined duration, therefore standardising the amount of time participants must hold sweet solutions in their mouth. The thermal stimulation paradigm allows a focus on C-fibre and Adelta-fibre pathways, which ascend the spinothalamic tract. Intersection of this pathway with brainstem centres such as the parabrachial nucleus and periaqueductal grey (Bernard *et al.*, 1995) offers a mechanism for the supraspinal modulation of nociception that we have previously proposed in the equivalent adult rat model of sucrose analgesia (Davies *et al.*, 2019). Whether mechanosensory information ascending via the dorsal-column pathways may additionally be intercepted by central modulatory mechanisms associated with reward is yet to be established.

***Reviewer: **Interestingly the article initially presents itself with the context that the human population widely seek sugar in times of ‘pain or discomfort seek solace in the form of sweet foods and drinks’. This statement highlights a phenomenon that is of significant importance. It is widely appreciated that pain is a fundamental physiological protective mechanism, as well as being a significant burden to the clinical setting inclusive of the patient, carer and healthcare systems. Additionally, during these times modern dietary composition and mental health are under the spotlight. Can this initial statement become expanded with additional literature to support how the authors see this study fitting into a wider research landscape? For example, is there data available to highlight increased sugar dietary consumption in pain patients or possible association with pain treatments or other consumed drugs? Consideration of the utilisation and benefit of using sweet taste would also be appreciated in the introduction to allow a greater audience to appreciate this work.*

**Author response: **Chronic pain has been associated with an increased prevalence of eating disorders in young people, and may include over-eating as well as under-eating conditions (Sim *et al.*, 2017). Obesity, for example, is often co-morbid with chronic pain; each condition negatively impacts the other, though the relationship is complex involving genetic and environmental factors (Okifuji & Hare, 2015). Anecdotal evidence suggests chronic pain sufferers may seek solace in the form of hedonic consumption (Amy Janke & Kozak, 2012), though again it is unclear whether this is related to an analgesic effect *per se*. We emphasise that the negative results of the current study do not allow us to speculate on the use or otherwise of sweet taste to engage such cognitive mechanisms at this time. However, we agree that this is an interesting area of study, and one that should benefit from further research into the neurobiology of the reward-pain axis and how the system may become imbalanced in injury or disease.

***Reviewer: **An aspect of the study is to elucidate whether there is a disconnect between heat pain perception and tolerance. The justification of this; both as anatomical and neuronal function was tentatively outlined in the introduction, an aspect of this was provided in the discussion as hedonic/reward via the limbic system. Could a detailed expansion of this be provided as currently ‘modulatory’ actions is used as a term and putative mechanisms of actions would be appreciated by the reader?*

**Author response: **Nociception, which encodes pain intensity, is discriminate, whereas pain tolerance has valency and can be affected by cognitive factors (Cimpean & David, 2019). Placing our results into context with the literature, we hypothesise that pain tolerance, and not merely pain intensity (threshold), may be preferentially affected by sweet consumption, as has been shown for cold pain tolerance (Lewkowski *et al.*, 2003). The wide distribution of pain processing throughout the brain and it’s separation into cognitive and emotional contexts provides numerous pathways for modulation of the pain experience (Tracey & Mantyh, 2007). We do not yet understand the neural circuits of sweet taste that appears able to suppress the valency of pain, but pathways related to reward, or the expectation of reward, as engaged by hedonic consumption (including sweet tasting food/drink) are likely to be involved. Further investigation of the potential central mechanisms of thermal sensory modulation by sucrose or other sweet tastes in humans awaits the further validation of assays for thermal pain tolerance (Lue *et al.*, 2018) that are ethically sound and sufficiently engage the relevant cognitive pathways.

The proposed methodology and general experimental design were appropriate, with overall structure well considered.

**Comments**

*1. In reference to the dose of sugar given; in the discussion it was highlighted that young adults in ref 26 were given a higher concentration of sucrose to induce analgesia. Could an aspect of discussion include reasoning around why a 10% dosing was utilised for this study, and how this differing dose may have implications in the data presented in this study. Also what dose is given in the clinic?*

**Author response: **We chose a concentration of 10% sucrose as this is the most commonly used concentration of sugar (typically sucrose) in sweetened beverages, including fruit juices (Ventura *et al.*, 2011). Concentrations of sucrose employed in studies of procedural analgesia in human neonates range from 7.5% to 50%, with 24% sucrose recommended prior to heel lancing and venepunctures (Stevens *et al.*, 2004). It is therefore possible that an analgesic effect could have been seen at higher concentrations of sucrose in our participants, as observed in the cold pressor task of pain tolerance (Lewkowski *et al.*, 2003).

It is noteworthy that two participants rated 10% sucrose as ‘unpleasant’. Between 0.25M and 0.5M sucrose (8.6% and 17.2%, respectively) is considered a flexion point in the ‘liking’ rating, such that concentrations of sucrose above this are characterised by a decrease in liking by ‘sweet dislikers’, which make up around 20% of adults aged 18-34 (Iatridi *et al.*, 2019). Indeed 10% sucrose (0.29M) is within the range of concentrations associated with the highest liking (0.21M-0.3M) (Iatridi *et al.*, 2019). Increasing the sweetness of the solution would therefore likely require stratification of the volunteer group by sweet preference to eliminate the confound of aversion in sweet ‘dislikers’. We have added a brief discussion of these limitations in the revised manuscript.

*2. 32°C used as a baseline why was this chosen? Room temp is usually 20-25. Does this negate detection thresholds?*

**Author response: **Although the ambient temperature of the environment was indeed between 20-25°C, a Peltier device set to this temperature would be perceived as ‘cold’; this is because the average skin temperature of the immediately exposed forearm is approximately 33°C (Barcroft & Edholm, 1946). Therefore 32°C was chosen to provide an imperceptible baseline for the temperature ramps, which we do not expect to interfere with the accurate detection of warm and thermal pain threshold. This baseline temperature is also standard for measuring warm detection and thermal pain thresholds in quantitative sensory testing protocols (Rolke *et al.*, 2006) **.** This has been clarified in the methods sections of the revised manuscript.

*3. 6 seconds between heating ramps? Is this sufficient time to provide recovery both dissipation of heat from the skin and also neuronal activity? For example skin temperature takes time to return to normal temperature post stimulation with varying temperature ramps whether hot or cold (McMullan 2004, Hulse 2012). Additionally, how the sensory afferents respond to these ‘rapid fire’ temperature spikes i.e. induction of nociceptor sensitisation/desensitisation.*

**Author response: **Heat ramps were performed in pairs with short (6 s) intervals: Warm detection thresholds were measured on the first ramp, and heat pain thresholds were measured on the second ramp. However, to clarify, a period of recovery of 2 min was then allowed before a repeat of the paired stimuli. We acknowledge that repeated short interval thermal stimulation could lead to sensitization, however we believe this was avoided by the longer interval between trials, thus allowing the skin temperature to return to normal. We do not expect a single warm ramp (to 39°C) to significantly affect subsequent thermal nociception (i.e. detection of painful heat); indeed the heat pain threshold measurements (average 43°C) were well within the range of healthy individuals (Rolke *et al.*, 2006) **. **We clarify these methodological details in the revised manuscript.

*4. Why was second dose of less concentrated sugar data not presented? Could this be included?*

**Author response: **As we describe in the paper, lower concentrations of sucrose (5%) and sucralose (0.008%) were tested for sweetness and pleasantness in a pilot study. As we found no difference in the ratings for either concentration, we continued the sensory testing with only the higher concentrations (10% sucrose and 0.016% sucralose equivalent), due to reasons outlined above.

*5. Figure 3 warm detection threshold test performed and mentioned but not presented. Could this be incorporated?*

**Author response: **For experiments on the effect of anticipation of sucrose only the heat pain thresholds were recorded. This has been clarified in the revised manuscript.

*6. A sentence in discussion could possibly be revised as it suggests that this article identifies an anti-nociceptive affect, which it did not. Recommendation to delete *italic *text: ‘It is therefore possible that purely anti-nociceptive effects of sucrose,* as measured by thermal sensitivity in our study *, are only present in neonatal and immature humans, which may account for, the lack of effect in this adult population’.*

**Author response: **We thank the reviewer for highlighting this grammatical error. This sentence has now been removed.

**References**

Amy Janke E & Kozak AT. (2012). "The more pain I have, the more I want to eat": obesity in the context of chronic pain. *Obesity (Silver Spring)* **20,** 2027-2034.

Anseloni VC, Ren K, Dubner R & Ennis M. (2005). A brainstem substrate for analgesia elicited by intraoral sucrose. *Neuroscience* **133,** 231-243.

Anseloni VC, Weng HR, Terayama R, Letizia D, Davis BJ, Ren K, Dubner R & Ennis M. (2002). Age-dependency of analgesia elicited by intraoral sucrose in acute and persistent pain models. *Pain* **97,** 93-103.

Barcroft H & Edholm OG. (1946). Temperature and blood flow in the human forearm. *The Journal of physiology* **104,** 366-376.

Bernard JF, Dallel R, Raboisson P, Villanueva L & Le Bars D. (1995). Organization of the efferent projections from the spinal cervical enlargement to the parabrachial area and periaqueductal gray: a PHA-L study in the rat. *J Comp Neurol* **353,** 480-505.

Cimpean A & David D. (2019). The mechanisms of pain tolerance and pain-related anxiety in acute pain. *Health Psychol Open* **6,** 2055102919865161.

Davies AJ, Kim D, Park J, Lee JY, Vang H, Pickering AE & Oh SB. (2019). Hedonic drinking engages a supraspinal inhibition of thermal nociception in adult rats. *Pain* **160,** 1059-1069.

Iatridi V, Hayes JE & Yeomans MR. (2019). Quantifying Sweet Taste Liker Phenotypes: Time for Some Consistency in the Classification Criteria. *Nutrients* **11**.

Lewkowski MD, Ditto B, Roussos M & Young SN. (2003). Sweet taste and blood pressure-related analgesia. *Pain* **106,** 181-186.

Lue YJ, Wang HH, Cheng KI, Chen CH & Lu YM. (2018). Thermal pain tolerance and pain rating in normal subjects: Gender and age effects. *Eur J Pain* **22,** 1035-1042.

Okifuji A & Hare BD. (2015). The association between chronic pain and obesity. *J Pain Res* **8,** 399-408.

Rolke R, Baron R, Maier C, Tolle TR, Treede RD, Beyer A, Binder A, Birbaumer N, Birklein F, Botefur IC, Braune S, Flor H, Huge V, Klug R, Landwehrmeyer GB, Magerl W, Maihofner C, Rolko C, Schaub C, Scherens A, Sprenger T, Valet M & Wasserka B. (2006). Quantitative sensory testing in the German Research Network on Neuropathic Pain (DFNS): standardized protocol and reference values. *Pain* **123,** 231-243.

Sim LA, Lebow J, Weiss K, Harrison T & Bruce B. (2017). Eating Disorders in Adolescents With Chronic Pain. *J Pediatr Health Care* **31,** 67-74.

Stevens B, Yamada J & Ohlsson A. (2004). Sucrose for analgesia in newborn infants undergoing painful procedures. *Cochrane Database Syst Rev* **,** CD001069.

Tracey I & Mantyh PW. (2007). The cerebral signature for pain perception and its modulation. *Neuron* **55,** 377-391.

Ventura EE, Davis JN & Goran MI. (2011). Sugar content of popular sweetened beverages based on objective laboratory analysis: focus on fructose content. *Obesity (Silver Spring)* **19,** 868-874.
